# COVID-19 Vaccine Effectiveness among Patients with Psoriatic Disease: A Population-Based Study

**DOI:** 10.3390/vaccines12050453

**Published:** 2024-04-24

**Authors:** Tal Gazitt, Lihi Eder, Walid Saliba, Nili Stein, Ilan Feldhamer, Arnon Dov Cohen, Devy Zisman

**Affiliations:** 1 Carmel Medical Center, Rheumatology Unit, Haifa 3436212, Israel; devyzi@clalit.org.il; 2 University of Washington Medical Center, Seattle, DC 98195, USA; 3 Ruth and Bruce Rappaport Faculty of Medicine, Technion, Haifa 31096, Israel; saliba_wa@clalit.org.il; 4 Department of Medicine, Women’s College Hospital, University of Toronto, Toronto, ON M5S 3H2, Canada; lihi.eder@wchospital.ca; 5 Department of Community Medicine and Epidemiology, Carmel Medical Center, Haifa 3436212, Israel; stein_nili@clalit.org.il; 6 Chief Physician’s Office, Central Headquarters, Clalit Health Services, Tel Aviv 67754, Israelarcohen@clalit.org.il (A.D.C.); 7 Siaal Research Center for Family Medicine and Primary Care, Faculty of Health Sciences, Ben-Gurion University of the Negev, Beer-Sheva 84105, Israel

**Keywords:** COVID-19 vaccine effectiveness, SARS-CoV-2, psoriatic arthritis, psoriasis, psoriatic disease

## Abstract

Limited information is available on the effectiveness of COVID-19 vaccination in patients with psoriasis and psoriatic arthritis (psoriatic disease (PsD)). The objective of our research was to assess the effectiveness of mRNA COVID-19 vaccination in preventing SARS-CoV-2 positivity and severe infection in a cohort of patients with PsD and the association of immunosuppressants on SARS-CoV-2 infection-related outcomes from December 2020 to December 2021. Vaccine effectiveness was assessed in a matched nested case control study using conditional logistic regression adjusted for demographics, comorbidities and immunosuppressant use. Study outcomes included SARS-CoV-2 positivity and severe COVID-19 (moderate-to-severe COVID-19-related hospitalizations or death). At least one dose of mRNA COVID-19 vaccine was associated with reduced risk of SARS-CoV-2 positivity and severe COVID-19 (OR = 0.41 (95% CI, 0.38–0.43) and OR = 0.15 (95% CI, 0.11–0.20), respectively). A more significant effect was found among patients who received three vaccines doses compared with those who did not receive any (OR (for positive SARS-CoV-2) = 0.13 (95% CI, 0.12–0.15) and OR (for severe disease) = 0.02 (0.01–0.05)). Etanercept and methotrexate were associated with higher risk of SARS-CoV-2 positivity (1.58 (1.19–2.10), *p* = 0.001 and 1.25 (1.03–1.51), *p* = 0.03, respectively). In conclusion, our results show that mRNA COVID-19 vaccines are effective in reducing both infection and severe COVID-19-related outcomes.

## 1. Introduction

The SARS-CoV-2-provoked COVID-19 pandemic has fostered the development and authorization of novel messenger RNA (mRNA) vaccines that have since proven to be immunogenic and effective in the immunocompetent population [1,2] Although patients with autoimmune inflammatory rheumatic diseases (AIIRD) were prioritized in terms of receiving COVID-19 vaccines, in order to mitigate SARS-CoV-2 infection risk [3], upon their initial approval, few data were made available on their efficacy and safety for this patient population. Only later did publications begin to show that these mRNA vaccinations are safe and effective in mixed populations of AIIRD patients [4,5,6], with very few studies focusing on specific sub-populations of AIIRD patients [7,8]. To date, there is a scarcity of data [9,10] that exists on the effectiveness of mRNA COVID-19 vaccines among patients with psoriasis (PsO) and/or psoriatic arthritis (PsA), collectively termed psoriatic disease (PsD). These patients are treated with a variety of immunosuppressive medications, including conventional, biologic, and targeted-synthetic disease-modifying anti-rheumatic drugs (c/b/ts DMARDs). In order to address this knowledge gap, the objective of our study was to assess the effectiveness of the BNT162b2 mRNA vaccine in preventing SARS-CoV-2 infections and COVID-19-related hospitalization and death in patients with PsD, and the effect of immunosuppressive medications on these COVID-19-related outcomes. This is of particular relevance given the recent rise in COVID-19 cases (World Health Organization (WHO) Coronavirus (COVID-19) Dashboard, https://covid19.who.int (accessed on 1 March 2024)).

## 2. Materials and Methods

### 2.1. Source of Data

This retrospective cohort study is based on the database of Clalit Health Services (CHS), the largest health care provider in Israel, which was described in detail in our previous study [11]. Briefly, this database includes information on approximately 4.7 million members, constituting ~56% of the population in Israel. The CHS membership is composed of individuals of a widely diverse geographic distribution, with different ethnicities and from all socioeconomic backgrounds across Israel, with all members having equal access to the same uniform medical benefits granted by the Israeli National Healthcare Plan, as mandated by the Israeli National Health Insurance Law (1995). CHS maintains a database that receives information updated continuously from pharmaceutical, medical, and administrative operating systems. Diagnoses are captured in the registry by diagnosis-specific algorithms, using the International Classification of Diseases 9th Revision (ICD-9) code reading, text editing, laboratory test results, and disease-specific drug usage. The first record of each data source is kept in the database, and the earliest recorded date, from any source, is used to define the starting date of the diagnosis. Medications dispensed are coded according to the Anatomical Therapeutic Chemical (ATC) classification. The database was designed for the purposes of administrative and clinical management and is available for use in epidemiological studies. The validity of selected disease diagnoses in the CHS database was found to be high in previous studies; specifically, the algorithm used to retrieve PsA patients (ICD-9 code 696.0) has been previously validated by our group by reviewing patient medical charts from community clinics and hospital discharge summaries, and was found to have high sensitivity (88.7%), specificity (88.1%), and positive predictive value (90.5%) [12,13]. The diagnosis of PsO (ICD-9 code 696.1) was also previously validated in a study reviewing medical charts from dermatology clinics and patient hospital discharge summaries [13].

Since the start of the COVID-19 pandemic, the Israeli Ministry of Health (MOH) has been collecting all COVID-19-related data and activities and has placed it into a national database. Among these activities are vaccination dates, active surveillance for all laboratory-confirmed SARS-CoV-2 infections with mandatory daily reporting of PCR and antigen results, and active surveillance of COVID-19-associated hospitalizations via daily updates from all hospitals, including daily status definitions during hospitalization. The collected data are transferred daily to the healthcare providers and also appear within the CHS database.

This study was approved by the Institutional Review Board of Carmel Medical Center (CMC-0014-14). Requirement for individual patient consent forms was waived due to the retrospective, observational nature of the study.

### 2.2. Study Population

All adult members of CHS aged 18 years or older with previous diagnosis of PsD prior to cohort entry date of 19 December 2020 (date of first dose of mRNA COVID-19 vaccine administration), were included in this retrospective study. The patients were followed from study entry date until SARS-CoV-2 infection and pre-defined COVID-19-related outcomes (discussed below), death, or study end date of 31 December 2021, whichever came first.

To assess the effectiveness of the BNT162b2 mRNA COVID-19 (Pfizer) vaccine in this patient population, we conducted a nested case–control study in which we identified all new cases of SARS-CoV-2 positivity among patients with PsD during the study period. For each SARS-CoV-2 positive case, (ascertained by SARS-CoV-2 polymerase chain reaction (PCR) positive test), which also served as the index date, we randomly selected four controls with PsD, who did not incur SARS-CoV-2 infection and were matched by age, sex, ethnicity and index date using risk set sampling. We then evaluated whether each SARS-CoV-2-positive patient had prior exposure to 1, 2, or 3 doses of mRNA COVID-19 vaccine at least two weeks prior to the index date. Similarly, to analyze risk factors for severe COVID-19-related outcomes (defined below), we performed a matched nested case–control study within the PsD cohort. All severe COVID-19 cases were identified from among the PsD patients and were matched, at a 1:4 ratio by sex, age, and ethnicity, to patients without severe disease.

### 2.3. Study Variables and Data Collection

For each patient, the following data were retrieved from the CHS database: demographic variables including age, sex, and ethnicity (Orthodox Jewish, secular Jewish, or Arab); socioeconomic status (SES) based on the SES score assigned to clinic neighborhoods as defined by the Israeli Central Bureau of Statistics; body mass index (BMI); presence of selected chronic comorbidities diabetes; ischemic heart disease (IHD); hypertension (HTN); hyperlipidemia (HLD); chronic renal failure (CRF); cerebrovascular accident (CVA); chronic obstructive pulmonary disease (COPD); and malignancy. Medication use during the four months preceding the index date was assessed, with all relevant medications approved both for PsO and PsA in Israel, including cDMARDs, bDMARDs, and tsDMARDs. The cDMARDs included acitretin, cyclosporine A, hydroxychloroquine, leflunomide, sulfasalazine, as well as methotrexate (MTX), which was analyzed separately, and apremilast, considered a small molecule inhibitor (“other immune-modulating therapy”) and included in the cDMARD medication category in our analysis. The bDMARDs included etanercept, which was analyzed separately from the monoclonal anti-TNF-α agents infliximab, adalimumab, golimumab, and certolizumab pegol; the IL-23 inhibitors risankizumab and guselkumab as well as the IL-12/23 inhibitor ustekinumab; and the IL-17 inhibitors secukinumab and ixekizumab. The tsDMARDs included the Jak inhibitors (Jak-I) tofacitinib and upadacitinib. Glucocorticosteroid (GC) use was defined as a minimum of one filled prescription of ≥5 mg of prednisone in the four months prior to index date.

### 2.4. Study Outcomes

The effectiveness of mRNA COVID-19 vaccinations was assessed in relation to the following two COVID-19-related outcomes: (1) first SARS-CoV-2 PCR test positivity; (2) severe SARS-CoV-2 infection, defined as moderate-to-severe COVID-19-related hospitalization (a hospitalization that was reported to the Israeli MOH as a hospitalization of a SARS-CoV-2 infected individual) or death (a death reported to the Israeli MOH as related to SARS-CoV-2 infection).

### 2.5. Statistical Analysis

In this study, descriptive statistics are reported with continuous variables summarized as mean ± standard deviation (SD) while categorical variables are presented as frequencies and proportions. Comparisons of baseline characteristics between PsA and PsO patients were undertaken using Chi square test or Fisher’s exact test, as appropriate. Continuous variables were compared using independent Student’s *t*-test.

Differences in baseline demographics and clinical characteristics between cases with SARS-CoV-2-positivty and their matched controls, and between cases with moderate/severe COVID-19 and their matched controls were compared using univariate and multivariate conditional logistic regression.

Vaccine effectiveness among patients with PsD was assessed using multivariable conditional logistic regression to estimate the odds ratio (OR) for the association between mRNA COVID-19 vaccine and the two main outcomes: SARS-CoV-2 positivity or moderate/severe COVID-19. mRNA COVID-19 vaccine was examined in two separate models, as a binary variable and as vaccine dose. In both models, we used no vaccination as a reference category.

Statistical analyses were performed using SAS version 9.4 (SAS Institute Inc., Cary NC, USA) and SPSS version 28 (IBM Corp. Released 2022. IBM SPSS Statistics for Windows, version 28.0, 2022, Armonk, NY, USA). For all analyses, *p* < 0.05 (for the 2-tailed tests) was considered statistically significant.

## 3. Results

**Figure 1:**
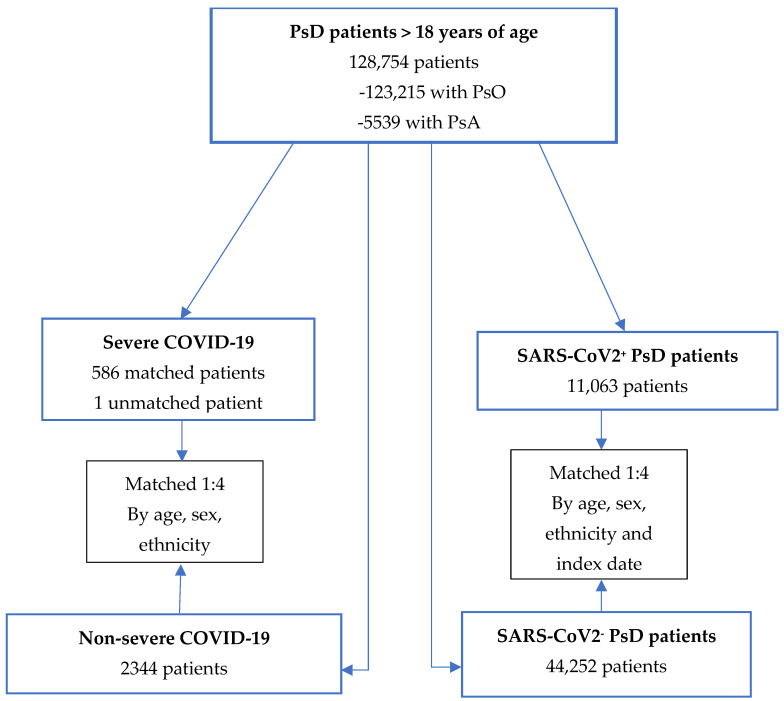
Study design. Abbreviations: PsA = psoriatic arthritis, PsD = psoriatic disease, PsO = psoriasis.

At study entry, 19 December 2020, the PsD cohort included in this study consisted of 128,754 individuals, of whom 123,215 were with PsO and 5539 with PsA (Figure 1). The average age of these patients was 52.7 ± 18.3 in the PsO group and 58.3 ± 15.0 in the PsA group, with 51.1% female in the PsO group and 54.3% in the PsA group (Table 1).

### 3.1. Risk Factors for SARS-CoV-2 Positivity and COVID-19 Severity

Of the 128,754 PsD patients enrolled in the study from 19 December 2020 until 31 December 2021, 11,066 (8.59%) individuals were SARS-CoV-2 positive. Three patients remained unmatched, so 11,063 patients were matched to 44,252 PsD patients who were negative for SARS-CoV-2 infection as controls. Out of all of the PsD patients, there were 587 (0.46%) patients who developed severe COVID-19, 586 of which were successfully matched to PsD patients without severe disease. Only one patient remained unmatched. Male patients made up the majority of severe COVID-19 patients (*n* = 340 (58.0%)).

In our study, we found that risk factors for SARS-CoV-2 positivity in PsD patients following multivariate analysis included low SES, obesity, past history of CVA, systemic use of GC, and hospitalization within the preceding year (Table 2A). We found that low SES, obesity, diabetes, previous CVA, CKD, COPD, use of systemic GC, and hospitalization within the previous year all contributed to risk of severe COVID-19 (Table 2B). PsA status was not associated with higher risk of SARS-CoV-2 positivity (Table 2A) or severe COVID-19-related outcomes (Table 2B) compared with patients with PsO alone. Notably, multivariate statistical models taking into account immunosuppressive medications did not alter these results. Among immunosuppressants used, etanercept and MTX were associated with higher risk of SARS-CoV-2 infection (1.58 (1.19–2.10), *p* = 0.001 and 1.25 (1.03–1.51), *p* = 0.03, respectively). No immunosuppressive agent was associated with severe COVID-19 (Table 2B).

### 3.2. Vaccine Effectiveness in PsD Patients

Among the 11,063 matched SARS-CoV-2-positive cases, mRNA COVID-19 vaccine was administered to 5410 (48.9%) patients compared with a total of 26,779 (60.5%) SARS-CoV-2 negative cases (Table 3). Our results show that at least one dose of mRNA COVID-19 vaccine was effective in preventing the disease, OR = 0.41 (0.38–0.43), compared to no vaccine administration (Table 4). An effective association was also shown in each vaccine dose (OR = 0.46 (0.42–0.51) for dose 1, OR = 0.46 (0.43–0.49) for dose 2, and OR = 0.14 (0.12–0.16) for dose 3) (Table 4).

Among severe COVID-19 cases, mRNA COVID-19 vaccine was administered to 208 patients (35.5%) patients compared with a total of 1467 (62.6 %) among the matched controls (Table 3). The results show that at least one dose of mRNA COVID-19 vaccine was effective in preventing severe disease (OR = 0.15 (0.11–0.20)) compared with no vaccine administration (Table 4). The mRNA COVID-19 vaccine was found to be effective in each vaccine dose with a dose-response effect (OR = 0.26 (0.17–0.40) for dose 1, OR = 0.14 (0.09–0.21) for dose 2 and OR = 0.03 (0.01–0.06) for dose 3) (Table 4).

## 4. Discussion

The SARS-CoV-2-provoked COVID-19 pandemic fostered the development and authorization of novel mRNA vaccines with little information available regarding the effectiveness of these vaccinations in specific sub-populations of AIIRD patients, including patients with PsD. Similar to previous studies [14,15,16], we found that male sex, lower SES, higher BMI, various CVD-related co-morbidities, such as past history of CVA, as well as prior hospitalization or exposure to systemic GC, all increase the risk of SARS-CoV-2 infection, and that these factors as well as COPD, CKD, and diabetes all increase the risk of severe COVID-19-related outcomes. These data are in accordance with data from the PsoProtect and Global Rheumatology Alliance physician-reported registries [10] on PsA and PsO showing that male sex, higher comorbidity burden, and GC intake are associated with COVID-19 severity.

Notably, within our PsD patient population, despite the high prevalence of cardiovascular-disease related risk factors which contribute to worse COVID-19-related outcomes and which are prevalent among patients with PsD [17,18], we found that even a single dose of mRNA COVID-19 vaccine was effective in reducing SARS-CoV-2 infection and severe COVID-19-related outcomes in this patient population. This finding is of significance, especially considering some recent studies showing reduced humoral response to the BNT162b2 mRNA COVID-19 vaccine in the form of reduced anti-SARS-CoV-2 IgG neutralizing antibodies in patients with autoimmune inflammatory rheumatic diseases (AIIRD) [19,20,21,22]. Among these studies, in our review of the literature, we found a single study on PsA patients that also shows reduced immune responsiveness in this cohort compared with healthy healthcare workers [9]. Indeed, most studies carried out to date have focused on the serologic response to mRNA COVID-19 vaccines in AIIRD patients, mostly in relation to healthy controls. These studies were mostly based on a heterogenous AIIRD patient population, including patients with inflammatory arthritis, connective tissue diseases, vasculitis, and even with patients with non-rheumatic diseases, such as inflammatory bowel disease (IBD). In our review of the literature, we found very few population-based studies like our study, which was carried out to evaluate the real-life effectiveness of BNT162b2 mRNA COVID-19 vaccine in a specific AIIRD patient population. One retrospective, population-based study by Widdifield et al., evaluating mRNA-based (BNT162b2 or mRNA-1273) COVID-19 vaccine effectiveness, demonstrated a reduction in both SARS-CoV-2 infection and severe COVID-19-related outcomes, but this, too, was in a heterogenous Canadian AIIRD patient population with rheumatoid arthritis, ankylosing spondylitis, psoriasis, and IBD in Ontario [5]. While we were unable to find large population-based studies focusing specifically on mRNA vaccine efficacy in PsD patients, a meta-analysis of several studies on systemic lupus erythematosus (SLE) also demonstrated mRNA COVID-19 vaccine efficacy [23].

In evaluating the effect of immunosuppressive medications on the effectiveness of mRNA COVID-19 vaccines, most studies thus far have again focused on the serologic response to vaccination. Generally, they have not shown differences in antibody responses between disease groups or overall immunomodulatory therapy categories [6], although patients on treatment regimens including mycophenolate mofetil (MMF) or rituximab (RTX) were seen to be less likely to develop antibody responses [4,24,25,26]. Our results, showing that the use of biologic therapy, including anti-TNF-α agents, IL-17 blockers and anti-IL-23 agents, did not increase risk of SARS-CoV-2 infection or COVID-19 disease severity in PsD patients, are in line with these studies, including the study by Benucci et al. on the humoral response to mRNA BNT162b2 vaccine in PsA patients [9]. However, unlike this study, which included only 10 patients on MTX, our results do associate MTX exposure with increased risk of SARS-CoV-2 infection. In line with our results is the finding by Furer et al. [4], revealing humoral response to mRNA vaccination to be mildly impaired by anti-TNF-α agents only when they were co-administered with MTX. Similar results are shown in a study by Frommert et al. [21] with lower seroconversion response to various types of homologous or heterologous COVID-19 vaccine regimens among AIIRD patients treated with agents such as MTX, MMF, RTX, or Jak-I, but not when treated with anti-TNF-α agents. Specifically, the effect of MTX on BNT162b2 mRNA COVID-19 vaccine responsiveness in a cohort of rheumatoid arthritis (RA) and a cohort of PsD patients was studied by Haberman et al. [27], showing both reduced humoral and cellular immunity in response to this agent. Such findings form the basis of the American College of Rheumatology (ACR) recommendations to withhold MTX for one-to-two weeks following vaccinations, including COVID-19 [28], in order to increase vaccine efficacy in AIIRD patients.

While our study did not analyze immune seroconversion in response to mRNA COVID-19 vaccination, we also found increased risk of acquiring SARS-CoV-2 infection in PsD patients treated specifically with the TNF-α inhibitor etanercept. Unlike our study, other studies did not carry out separate analyses on the effect of individual anti-TNF-α agents on the effectiveness of COVID-19 vaccination, with some data compiled separately and solely on infliximab of the five anti-TNF-α agents used in rheumatology practice today, meaning that the safety signal on etanercept relative to other TNF-α inhibitors might have been missed in other studies. One putative reason for our findings, related specifically to etanercept use, might lie in the difference in mechanism of action of etanercept as a soluble TNF-α receptor blocker relative to the monoclonal anti-TNF-α agents. Alternatively, these findings might be related to the selective administration of etanercept to patients with longstanding PsD with recurrent infections or comorbidities who are prescribed an agent with a shorter half-life, like etanercept. In this case, the increase in risk of SARS-CoV-2 infection might be related to this cohort of patients being more vulnerable to infection, and not to etanercept itself.

Notably, we did not find any immunosuppressive agent with which to increase the risk of severe COVID-19. This finding concurs with other studies [29,30] on AIIRD patients that have focused mostly on patients with IBD and which found TNF-α inhibitor monotherapy to be associated with a lower risk of adverse COVID-19-related outcomes when compared with other commonly prescribed immunomodulatory treatment regimens. In fact, anti-TNF-α therapy has been shown to have a protective effect in suppressing the maladaptive systemic inflammatory response that often characterizes severe COVID-19 [31], with several case series reporting more favorable COVID-19-related outcomes among both children and adults with COVID-19-related hyperinflammatory syndrome treated by the monoclonal anti-TNF-α agent infliximab [32,33]. These findings are supported by a study [34] showing that hospitalized COVID-19 patients have elevated TNF-α concentrations during hospital admission, and that these are associated with organ damage and worse COVID-19-related outcomes, thus explaining the improved outcome with TNF-α blockade.

Possible limitations in our study include the general limitations of administrative database research, such as lack of clinical data on PsD disease activity, which might affect the choice of immunosuppressive agents used, and a lack of data on the doses of immunosuppressive medications, which may affect the risk of infection and the potential presence of unmeasured confounders. In addition, while we did not find any association between any immunosuppressive agents and COVID-19 severity, our study may have not had enough statistical power to detect such a risk due to the small number of cases with some of the specific immunosuppressive medications. Despite the aforementioned limitations, our study is founded on a database of 4.7 million individuals featuring data on patients with PsD in real life. Importantly, our study shows that the novel mRNA vaccines are effective in reducing SARS-CoV-2-related infection and hospitalization even in immunosuppressed populations with comorbidities that typically increase infection risk, such as those found in the PsD patient population.

## 5. Conclusions

In summary, in our population-based study, we found that mRNA COVID-19 vaccines are effective in reducing both infection and severe COVID-19-related outcomes in the specific population of PsD. Overall, these results are reassuring, especially in light of the increased prevalence of SARS-CoV-2 infections and the high prevalence of cardiovascular disease-related comorbidities found among patients with PsD, which are known risk factors for more severe COVID-19-related outcomes.

## Figures and Tables

**Table 1:** Characteristics of the PsD population at study entry.

	PsD	PsO	PsA	*p*-Value
**PsD patients** (N patients)	128,754	123,215	5539	
**Age** (Mean ± SD) Median (IQR)	53.0 ± 18.2 59.8 (46.4–69.0)	52.7 ± 18.3 52.1 (37.4–67.7)	58.3 ± 15.0 59.9 (46.7–69.4)	*p* < 0.001
**Sex (Female)** N (% within cohort)	62,815 (48.8)	62,930 (51.1)	3009 (54.3)	*p* < 0.001
**Ethnicity** N (% within cohort) Jewish	108,846 (84.5)	104,048 (84.4)	4798 (86.6)	*p* < 0.001
Arab	19,908 (15.5)	19,167 (15.6)	741 (13.4)	
Socio-economic status (SES) * N (% within cohort) Low	40,449 (31.4)	38,710 (31.4)	1739 (31.4)	*p * = 0.893
Medium	52,370 (40.7)	50,122 (40.7)	2248 (40.6)	
High	34,920 (27.1)	33,407 (27.1)	1513 (27.3)	
Missing	1015 (0.8)	976 (0.8)	39 (0.7)	
Obesity * (BMI ≥ 30 (kg/m^2^) (N, % within cohort)	36,972 (28.7)	34,870 (28.3)	2102 (37.9)	*p* < 0.001
**Comorbidity** (N, % within cohort) Malignancy	14,632 (11.4)	13,596 (11)	1036 (18.7)	*p* < 0.001
Diabetes	24,578 (19.1)	23,064 (18.7)	1514 (27.3)	*p* < 0.001
HLD	62,872 (48.8)	59,374 (48.2)	3498 (63.2)	*p* < 0.001
IHD	13,798 (10.7)	12,991 (10.5)	807 (14.6)	*p* < 0.001
HTN	36,923 (28.7)	34,754 (28.2)	2169 (39.2)	*p* < 0.001
CKD	11,328 (8.8)	10,678 (8.7)	650 (11.7)	*p* < 0.001
CVA	5528 (4.3)	5189 (4.2)	339 (6.1)	*p* < 0.001
COPD	4709 (3.7)	4403 (3.6)	306 (5.5)	*p* < 0.001

Abbreviations: BMI = body mass index, CKD = chronic kidney disease, COPD = chronic obstructive pulmonary disease, CVA = cerebrovascular accident, HLD = hyperlipidemia, HTN = hypertension, IHD = ischemic heart disease, N = number, PsA = psoriatic arthritis, PsD = psoriatic disease, PsO = psoriasis, Ref = reference, SD = standard deviation. * Presented data for percentages for socioeconomic status and BMI were calculated from the total number of the available data.

**Table 2:** (**A**) Characteristics of PsD patients’ cases and controls included in nested case–control study and assessment of risk factors for SARS-CoV-2 positivity. (**B**) Characteristics of PsD patients’ cases and controls included in nested case–control study and assessment of risk factors for severe COVID-19.

(**A**)					
		**SARS-CoV-2^+^ Cases**	**Matched Controls**	**Adjusted ^a^** **OR (95% CI)**	***p*-Value**
**PsD patients** N patients (%)		11,063	44,252		
**PsA vs. PsO**		448 (4.0)	1730 (3.9)	0.99 (0.89–1.10)	0.83
**Age** (Mean ± SD)		46.5 ± 16.6	46.7 ± 16.6	N.A	N.A
**Sex** N patients (%)	Male	5114 (46.2)	20,456 (46.2)	N.A	N.A
**Ethnicity**	Jews	8907 (80.5)	35,628 (80.5)	N.A	N.A
**Socio-economic status (SES) ^b^** N patients (%)	Low	4553 (41.2)	14,737 (33.3)	Reference	<0.001
	Medium	4242 (38.3)	17,382 (39.3)	0.71 (0.57–0.87)	
	High	2145 (19.4)	11,769 (26.6)	0.56 (0.45–0.69)	
**BMI ^b^** (kg/m^2^) N patients (%)	BMI < 25	2441 (22.1)	10,470(23.7)	Reference	<0.001
	BMI ≥ 25 < 30	2777 (25.1)	10,733 (24.3)	1.13 (1.06–1.21)	
	BMI ≥ 30	2557 (23.1)	9230 (20.9)	1.19 (1.12–1.28)	
**Comorbidity** N patients (%)	Malignancy	843 (7.6)	3465 (7.8)	0.96 (0.88–1.05)	0.35
	Diabetes	1632 (14.8)	6110 (13.8)	1.08 (1.00–1.16)	0.051
	IHD	778 (7.0)	2917(6.6)	1.06 (0.96–1.17)	0.22
	CVA	380 (3.4)	1188 (2.7)	1.19 (1.05–1.36)	0.007
	CKD	821 (7.4)	3030 (6.8)	1.05 (0.97–1.15)	0.24
	COPD	172 (2.9)	1476 (2.5)	1.03 (0.89–1.19)	0.68
	HTN	2180 (19.3)	8530 (19.3)	0.98 (0.91–1.06)	0.62
	HLD	4065 (36.7)	16,917 (38.2)	0.90 (0.85–0.95)	<0.001
**Systemic GC** N patients (%)		1165 (10.5)	4240 (9.6)	1.10 (1.02–1.18)	0.01
**Medications** N patients (%)	cDMARDS	195 (1.76)	808 (1.83)	0.99 (0.84–1.16)	0.88
	Anti-TNF-α ^c^	237 (2.1)	817 (1.8)	1.12 (0.93–1.35)	0.221
	Jak-I	11 (0.1)	35 (0.1)	1.20 (0.60–2.42)	0.61
	Anti-IL-23	50 (0.45)	233 (0.53)	0.89 (0.65–1.22)	0.46
	Anti-IL-17	61 (0.55)	287 (0.65)	0.85 (0.63–1.13)	0.26
	Etanercept	74 (0.67)	209 (0.47)	1.58 (1.19–2.10)	0.001
	MTX	157 (1.42)	513 (1.16)	1.25 (1.03–1.51)	0.03
**Hospitalization during the prior year** N patients (%)		1252 (11.3)	4181 (9.4)	1.180 (1.10–1.27)	<0.001
(**B**)					
		**Severe COVID-19 Cases**	**Matched Controls**	**Adjusted ^a^** **OR (95% CI)**	***p*-Value**
**PsD patients** N patients (%)		586	2344		
**PsA vs. PsO**		31 (5.3)	125 (5.3)	0.89 (0.56–1.41)	0.62
**Age** (Mean ± SD)		67.5 ± 16.1	67.6± 16.0	N.A	N.A
**Sex** N patients (%)	Male	340 (58.0%)	1360 (58.0)	N.A	N.A
**Ethnicity**	Jews	444 (75.8)	1776 (75.8)	N.A	N.A
**Socio-economic status (SES) ^b^** N patients (%)	Low	270 (46.1)	814 (34.7)	Reference	<0.001
	Medium	218 (37.2)	897 (38.3)	0.11 (0.04–0.29)	
	High	83 (14.2)	624 (26.6)	0.063 (0.02–0.18)	
**BMI ^b^** (kg/m^2^) N patients (%)	BMI < 25	86 (14.7)	526 (22.4)	Reference	0. 003
	BMI ≥ 25 < 30	188 (32.1)	796 (34.0)	1.42 (1.03–1.94)	
	BMI ≥ 30	228 (38.9)	672 (28.7)	1.79 (1.3–2.46)	
**Comorbidity** N patients (%)	Malignancy	139 (23.7)	471 (20.1)	1.28 (0.98–1.67)	0.066
	Diabetes	276 (47.1)	787 (33.6)	1.61 (1.26–2.06)	<0.001
	IHD	170 (29.0)	521(22.2)	1.06 {0.81–1.39)	0.68
	CVA	103 (17.6)	202 (8.6)	1.9 (1.39–2.6)	<0.001
	CKD	141 (24.1)	357 (15.2)	1.47 (1.12–1.93)	0.006
	COPD	81 (13.8)	173 (7.4)	1.81 (1.26–2.60)	0.001
	HTN	370 (63.1)	1236 (52.7)	1.27 (0.96–1.68)	0.093
	HLD	455 (77.6)	1667 (71.1)	1.19 (0.88–1.6)	0.26
**Systemic GC** N patients (%)		131 (22.4)	362 (15.4)	1.52 (1.16–1.99)	<0.002
**Medications** N patients (%)	cDMARDS	14 (2.39)	67 (2.9)	0.91 (0.46–1.79)	0.78
	Anti-TNF-α ^c^	5 (0.9)	41 (1.7)	0.45 (0.11–1.83)	0.27
	Jak-I	2 (0.34)	1 (0.04)	7.69 (0.58–101.5)	0.12
	Anti-IL-23	6 (1.02)	12 (0.5)	2.04 (0.64–6.46)	0.23
	Anti-IL-17	3 (0.51)	11 (0.47)	1.57 (0.39–6.37)	0.53
	Etanercept	1 (0.17)	14 (0.60)	0.42 (0.05–3.60)	0.43
	MTX	12 (2.05)	52 (2.22)	1.58 (0.73–3.41)	0.25
**Hospitalization during the prior year** N patients (%)		183 (31.2)	418 (17.8)	1.43 (1.1–1.87)	0.008

Abbreviations: Anti-IL (anti-interleukin). Anti-TNF-α = anti-tumor necrosis factor-α, BMI = body mass index, cDMARDs = conventional disease modifying anti-rheumatic drugs, CI = confidence interval, CKD = chronic kidney disease, COPD = chronic obstructive pulmonary disease, CVA = cerebrovascular accident, GC = glucocorticosteroids, HLD = hyperlipidemia, HR = hazard ratio, HTN = hypertension, IHD = ischemic heart disease, Jak-I = janus kinase inhibitors, MTX = methotrexate, N = number, N.A = not applicable, OR = odds ratio, PsA = psoriatic arthritis, PsD = psoriatic disease, Ref = reference, SD = Standard deviation, SES = socioeconomic status. (A) The SARS-CoV2 PCR positive PsD population included in the nested study and presented in this table was matched at a 1:4 ratio to PsD patients who were negative for severe COVID-19. (B) The PsD population with severe COVID-19 included in the nested study were identified from among the PsD patients (they all had positive SARS-CoV-2 PCR test) and were matched at a 1:4 ratio by sex, age, and ethnicity to patients without severe disease. ^a^ Adjusted odds ratio refers to the results of the multivariable analysis. ^b^ Presented data for percentages for socioeconomic status and BMI were calculated from the total number of the available data. ^c^ Anti-TNF-α agents included all monoclonal anti-TNF-α agents except for etanercept, which was analyzed separately.

**Table 3:** Association between COVID-19 vaccination status and SARS-CoV-2 positivity among patients with PsD.

	SARS-CoV-2 Positive Patients (%) *n* = 11,063	Matched Controls *n* = 44,252	* Crude OR (95% CI)	** Adjusted OR (95% CI)
No vaccine	5653 (51.1)	17,473 (39.5)	Ref	Ref
Dose 1	707 (6.4)	4223 (9.5)	0.45 (0.41–0.49)	0.46 (0.42–0.51)
Dose 2	4086 (36.9)	17,536 (39.6)	0.44 (0.41–0.47)	0.46 (0.43–0.49)
Dose 3	617 (5.6)	5020 (11.3)	0.13 (0.12–0.15)	0.14 (0.12–0.16)
Any vaccine dose	5410 (48.9)	26,779 (60.5)	0.39 (0.37–0.41)*	0.41 (0.38–0.43)

Abbreviations: CI = confidence interval, OR = odds ratio, PsD = psoriatic disease, Ref = reference. * Raw data were adjusted for age, sex, and ethnicity. ** Multivariate models were adjusted for age, sex, ethnicity, socioeconomic status, body mass index, tobacco use, diabetes mellitus, hypertension, hyperlipidemia, ischemic heart disease, previous cerebrovascular accident, chronic kidney disease, chronic obstructive pulmonary disease, malignancy, prior hospital admission within the past year prior to study entrance, and glucocorticosteroid use. In both models, no vaccination was used as a reference category.

**Table 4:** Association between COVID-19 vaccination status and COVID-19 severity among patients with PsD.

	Severe COVID-19 Patients (%) *n* = 586	Matched Controls *n* = 2344	* Crude OR (95% CI)	** Adjusted OR (95% CI)
No Vaccine	378 (64.5)	877 (37.4)	Ref	Ref
Dose 1	50 (8.5)	347 (14.8)	0.25 (0.17–0.37)	0.26 (0.17–0.40)
Dose 2	142 (24.2)	823 (35.1)	0.14 (0.10–0.20)	0.14 (0.09–0.21)
Dose 3	16 (2.7)	297 (12.2)	0.02 (0.01–0.05)	0.03 (0.01–0.06)
Any vaccine dose	208 (35.5)	1467 (62.6)	0.14 (0.11–0.18)	0.15 (0.11–0.20)

Abbreviations: CI = confidence interval, OR = odds ratio, PsD = psoriatic disease, Ref = reference. * Raw data were adjusted for age, sex, and ethnicity. ** Multivariate models were adjusted for age, sex, ethnicity, socioeconomic status, body mass index, tobacco use, diabetes mellitus, hypertension, hyperlipidemia, ischemic heart disease, previous cerebrovascular accident, chronic kidney disease, chronic obstructive pulmonary disease, malignancy, prior hospital admission within the past year prior to study entrance, and glucocorticosteroid use. In both models, no vaccination was used as a reference category.

## Data Availability

The data used and/or analyzed during the present study are available from the corresponding author on reasonable request.
